# Canonical circuits of the cerebral cortex as enablers of neuroprosthetics

**DOI:** 10.3389/fnsys.2013.00077

**Published:** 2013-11-08

**Authors:** Manuel F. Casanova

**Affiliations:** Gottfried and Gisela Kolb Endowed Chair in Psychiatry, University of LouisvilleLouisville, KY, USA

**Keywords:** minicolumns, microcircuits, cerebral cortex, neuroprosthetics

“…each technical advance over the past century has reaffirmed that repeated patterns of structure and function are seen at every level, from molecule to cell to circuit, and that many of these patterns are common across cortical areas and species. In this context, the concept of a canonical circuit, like the concept of hierarchies of processing, offers a powerful unifying principle that links structural and functional levels of analysis across species and different areas of the cortex”

(Douglas and Martin, 2010, p. 20).

The traditional microscopic assessment by a neuropathologist is usually accomplished by first examining sampled sections at low magnification looking for abnormalities of tissue characteristics and then at higher magnification for abnormalities in the morphometry of individual cellular elements. The presence of pathology is usually ascertained if cells are missing, reduced in size or exhibit aberrant staining properties. These cellular characteristics are not salient findings in many psychiatric conditions such as schizophrenia, autism and bipolar disorders. Given the large number of symptoms that are localizable to the central nervous system, the paradoxical absence of neuropathology in many psychiatric disorders makes us wonder whether we are missing some abnormalities. In other words, it is justifiable to consider whether pathology in these conditions escapes the level of resolution usually assessed by the neuropathologist. Cerebral abnormalities in many psychiatric conditions may not be evident in single cells but rather in units of neurons working together as circuitry. In this regard it is tempting to suggest a chiasm between medical disciplines: that neuronal abnormalities define the pathology of neurological disorders (e.g., Alzheimer's or Parkinson's disease) while those involving circuitry define abnormalities in psychiatric disorders (e.g., autism, schizophrenia).

Some years ago the famous historian of science, Thomas S. Kuhn wrote a popular book and bestseller entitled “The Structure of Scientific Revolution” (Kuhn, 1970). In the book Kuhn made the case that in order for science to advance it needed a “paradigm shift,” a change in our way of thinking or in our approach to a problem. In the case of air travel, faster airplanes are not the product of bigger propeller engines that generate increasing thrust; rather, different engines and physical principles account for advancement in air travel. Many modern airplanes are propelled by a gas turbine while rocket engines use the thrust of their own combustion exhaust gas. In similar fashion, major advancements in science are not the result of gradual increments applying the same technology (e.g., bigger propeller engines for air travel), but respond to the introduction of a new way of thinking on an older problem.

For many decades neuroscience has been dominated by the cell theory of Schwann and Schleiden that argued in favor of the existence of a unique type of cell exemplifying the holistic properties of each individual organ. Acceptance of this reductionist approach has been promoted by the explanatory powers derived from the work of such an eminent neuroscientist as Theodor Meynert who used cellular details, as to both form and spatial organization, in order to parcellate the cerebral cortex. Santiago Ramón y Cajal extended this neuronocentric view by providing evidence that favored what would later on be called the “neuronal doctrine.” In essence Ramón y Cajal argued that the relationship between neurons was not one of continuity but of contiguity.

The existence of a generalizable neuron with a clear separation between its functional components is an oversimplification. Countless number of neurons exist within the brain differing from one another in terms of size, shape, location and neurotransmission. Contrary to Ramón y Cajal's law of dynamic polarization the dendrites of some neurons may occasionally generate an action potential and axons my bear receptive surfaces. Many neurons throughout the animal kingdom lack axons while others release their neurotransmitters through non-synaptic sites (Casanova, 2010). The plurality of neurons argues against their designation as “individual” elements of the brain (Casanova, 2010).

Although the anatomical evidence prevailed during the ensuing decades it flew against opposition emanating from physiological studies. Sir Charles Sherrington posited the interactive function of neural elements, both excitatory and inhibitory, the simplest of which was the reflex arc. Still, even within a reflex arc, simple actions require the coordinated efforts of many neurons: “The reflex arc consisting of just two neurons is an abstraction … Even in systems such as the monosynaptic myotatic reflex in mammals, in which there is one set of afferent and one set of efferent neurons, many neurons are involved” (Brown, 2001, p. 146).

Actions within the nervous system are the result of neuronal ensembles, not of single cellular entities. The concept of cell assemblies acting at any given moment within closed systems was popularized by Donald Hebb in his famous book “The Organization of Behavior” (Hebb, 1957). According to this view, groups of neurons are capable of acting as a closed system and, depending on functional requirements, its individuals components can participate in different cell assemblies. This interdependence of neurons is evident both *in vivo* as well as *in vitro*.

Cultured neurons are usually derived from stem cells that, depending on need, are later on differentiated into neurons, astrocytes or oligodendrocytes. In the case of neurons scientist can manipulate the culture media to enable cells to generate synapses and myelination. Still, the initial cellular density is critical to the survival of the colony. This factor, called the seeding density, usually varies between 80 and 300 cells/mm^2^. We can safely conclude that neurons can't survive and perform their function in isolation. According to Shepherd and Koch, “No matter how complicated a single neuron may be, it cannot play a role in the processing of information without interacting with other neurons” (Shepherd, 2004, p. 27).

It is clear that a single neuron does not represent the holistic properties of the brain nor can it represent the basic unit of function for this organ. Only networks of neurons achieve this distinction. According to the work of the American psychologist and behaviorist Karl Spencer Lashley these networks appear widely distributed throughout the brain. Lashley's work using rat's brains failed to localize the substratum of memory to a particular area of the brain. In effect, he was the first person to spouse, based on his experimental work, the principle of equipotentiality. This principle antedated claims from modern neuroplasticity experiments that if certain parts of the brain are damaged, other regions may take over the function. Equipotentiality in this regard suggests the presence of circuitry capable of performing the same generic operations throughout different parts of the cortex.

The existence of repetitive circuits carrying generic types of operations within the cerebral cortex has been well discussed within the field of neuroanatomy. The nomenclature for this reiterative circuit has shifted through the decades being called either a basic, local or canonical circuit by different authorities (Shepherd, 1974, 1978; Rakic, 1975; Douglas and Martin, 1991). Lorente de No was the first researcher to propose the existence of vertically oriented cellular elements within the cerebral cortex that conjointly acted as a circuit. Lorente de No described these vertically arranged cellular aggregates as follows: “All the elements of the cortex are represented in it, and therefore it may be called an elementary unit, in which, theoretically, the whole process of transmission of impulses from the afferent fiber to the efferent axon may be accomplished” (Lorente de No, 1938). Not surprisingly Lorente de No was honored by the American Philosophical Society with the first Karl Spencer Lashley Award in 1959.

It is noteworthy that most of Lashley's work was performed while at the Johns Hopkins School of Medicine. It was in this academic setting that Stephen Kuffler employed microelectrodes to investigate receptive fields of retinal ganglion cells and their center-surround inhibition. Mountcastle and colleagues refined Kuffler's technique in order to proclaim the landmark discovery of functional cortical columns in the somatosensory cortex of cats and monkeys. The columnar organization proclaimed by Mountcastle was confirmed in two early experiments wherein slanted penetrations, i.e., an angle of 45° to the surface of the brain, demonstrated changes in modality as the microelectrode transversed neighboring tissue (Mountcastle, 1957).

Mountcastle's work indicated the presence of vertically arranged cellular structures with similar electrophysiological properties in different parts of the brain. The findings suggested that the cerebral cortex was more homogenous in its function than previously thought. According to Bach-y-Rita, this meant that, “… any part of the cortex should be able to process whatever electrical signals were sent to it, and that our brain modules were not so specialized after all” (Doidge, 2007, p. 18). Otto Creutzfeld believed that these repetitive neocortical microcircuits processed information in similar manner with the resultant output depending on both the source of information and modulatory influences peculiar to each brain region (Creutzfeldt, 1977). The seminal observations of Mountcastle's were later on expanded upon by two of his disciples: Apostolos Georgopolous and Michael Merzenich.

In the 1980s Apostolos Georgopolous used a population vector model to describe how groups of neurons act as voting circuits by using their firing rates as ballots to define the activity of individual components (Georgopolous et al., 1988). The final vote is tabulated by the vectorial sum of each cell's preferred orientation weighed by their firing rates. This model enabled the encoding of programs in monkeys that translated visual stimuli into reach direction.

Michael Merzenich used microelectrode techniques to show the rapidly changing nature of somatotopic and tonotopic maps in response to environmental exigencies. His notion about how the brain is capable of modifying itself (plasticity) enabled him to lead the cochlear implant team at UCSF (Merzenich et al., 1977). Merzenich's work has shown how artificial stimuli can be used to retrain cortical circuitry respective of brain region. In this regard the plasticity of the brain is dependent on the existence of “on-demand” event-based processing circuits. These cortical circuits are connected in a series of nested positive and negative feedback loops called repetitive or recurrent circuits (Douglas and Martin, 2010).

In recent years, Opris et al. (2012) implemented the use of a unique conformal multielectrode recording array to define the role of interlaminar circuitry within prefrontal cortex minicolumns in task-related target selection in nonhuman primates. Activation of innate prefrontal cortex minicolumns via the encoded interlaminar correlated firing sequences resulted in improved performance on trials where specific information was required depending on context (Opris et al., 2013). The results indicate interlaminar correlated firing during the decision phase of target selection and provide a direct demonstration of real-time minicolumnar processing during an executive function task. The discovery holds important promise for using minicolumnar circuitry in cognitive prosthetics.

In summary, brain plasticity holds the promise of restoring cognitive functions even when brain tissue has been lost. The use of neuroprosthetics is largely predicated on the existence of generic circuits which can be exapted by artificial stimuli, or in the case of cognitive neuroprosthetics, replaced by integrated circuits capable of processing stimuli in a manner similar to that normally done by the diseased area of the brain. In this regard, our ability to provide brain-machine interfaces is grounded on a large body of knowledge from pioneering anatomists and physiologists in regards to the microcircuitry of the brain.
